# Solving the Hospital Financial Crisis

**Published:** 1920-01-10

**Authors:** 


### The Hospital

The Workers' Newspaper of Administrative Medicine and Institutional Life, Administration, National Insurance and Health.

No. 1753, Vol. LXVII. SATURDAY, JANUARY 10, 1920. PRICE TWOPENCE

### SOLVING THE HOSPITAL FINANCIAL CRISIS.

When this number of The Hospital is published the Christmas holidays will have ended, and it will be the High privilege of all hospital people throughout Great Britain to awake, put their house in order, and begin to prepare with purposeful energy to take their part in the great effort which is to be made, everywhere throughout Great Britain, to put the voluntary hospitals on a sound financial basis, by June 30 next. Then the managers should be placed in a position to carry on successfully the active and increased work of every voluntary hospital of importance in each English, Welsh, and Scottish county.

During the recess another great centre of hospital work—Leeds—has been stimulated with hope and energy by the receipt of a very generous gift of £50,000 from Mr. Joseph Watson, of Linton Springs, Wetherby. Mr. Watson stipulates that £10,000 of this sum shall be invested and the proceeds used as a Nurses' Pension Fund, and that the balance of £40,000 shall be paid into the general funds of the Infirmary. He hopes that it may be possible to devote some part of the £40,000 towards the replacement of the £58,000 worth of investments sold to meet the extra expenditure occasioned by the war. Be this as it may, it should prove a great encouragement to the managers of every voluntary hospital throughout Great Britain to hear of Mr. Watson's splendid gift, and to remember that this is the sixth contribution of its kind within a few weeks, the others being £20,000 to Liverpool, £20,000 to Manchester, £20,000 to Guy's Hospital, Nottingham practically £100,000 as a War Memorial, and £20,000 for maintenance.

There is here a splendid opportunity for the profiteer and the wealthy in every county throughout England, Wales, and Scotland to see that the voluntary hospitals of each county without delay receive a pioneer gift of a large sum. To effect this it is essential that the hospital authorities should wake up everywhere throughout Great Britain. In each centre of population a committee should be organised, consisting of representative and able local men and women, a goodly number of whom should be recruited from the warriors and the noble women who have returned from the war, so that new blood may be introduced and all the necessary machinery got ready for a great effort throughout every district of Great Britain to awaken the interests of the 90 per cent. of the inhabitants, everywhere, who have heretofore taken little or no interest in the maintenance of the voluntary hospitals. They have yet to learn to appreciate the splendid work these hospitals do for the community. Once this movement is successfully launched, all classes will soon come to realise the large sum of money that is required to extend the areas of Hospital usefulness to a point, which will provide ample hospital accommodation for all who may be urgently in need of it.

The first and most important step is to recruit this new army of interested workers who, from their experience gained during the war, have become convinced of the splendid efficiency of this type of hospital under British administration. We regret that there are still a few hospitals with which we have communicated by letter, that have not yet shown any sign of interest or willingness to help in a movement to bring funds to their institution, and increase its power of succour to the full extent needed. We have in preparation an analysis of the institutions which have been communicated with, the answers which have been received, and the dumb districts which have institutions whose representatives appear to be buried in slumber. It would be a good thing if the giving public, and those who are conscious of the splendid work of the hospitals in every centre of population throughout England, Scotland, and Wales, would make it their business to call at their local hospital and ascertain for themselves whether they have received The Hospital's communication, and what steps, if any, have been taken or are contemplated with the view of joining the army of workers who are marshalling to organise methods, whereby the whole country may have its attention called to the present needs of the voluntary hospitals, and to have those needs explained and largely mitigated by the efforts of thousands of voluntary workers present amongst them, many of whom have worked so hard and so successfully for the Red Cross during the days of the war.

A further vital point is to receive evidence that every hospital of any importance throughout Great Britain is really awake to the need there is for a united effort, everywhere, to obtain considerable contributions from new sources of supply, by enlisting the interest and support of **the 90 per cent. of people who have yet to show an interest in the voluntary hospital.** We have confidence that the majority, if properly approached, will gladly lend a hand to the speedy accomplishment of the splendid work, through voluntary service, on the part of the healthy in aid of the sick, who need it as an urgent and vital necessity. It is essential that if possible bread-earners in every district of the country are to be equipped with health, that they shall make their efforts equal to the need, and worthy of the spirit which every intelligent and awakened man and woman should bring to the task.

It must be evident that, to succeed in the glorious task of putting the finances of the voluntary hospitals once and for all upon a sound basis, what is required is a long pull, a strong pull, and a pull all together, by the people in every district and centre where there are hospitals, for there evidence of the need will become manifest to the investigator. Then, we hope and believe, his or her whole-hearted co-operation and invaluable help as workers for the sick will be gladly given. Glasgow during Christmas time has given evidence of awakening. Preparations are in progress to enable the citizens to realise, that their great infirmaries cannot discharge their most Christian and humane functions, as the *Glasgow Herald* forcibly insisted on New Year's Day, unless benevolent people in their midst will adhere faithfully to the principle of "generous giving, or alternatively will submit themselves to a *régime* that will ensure the automatic and adequate sustentation of these indispensable institutions."

The *Glasgow Herald* properly insists that by some means or other the greatest of Glasgow charities must be rescued from their present condition of impoverishment and consequent enfeeblement and impending disablement. This view is shared throughout the United Kingdom by every intelligent man and woman who has a mind to realise the facts and a heart to feel the urgent need for help. If it be true that 10 per cent. of the citizens by their individual efforts and public spirit have heretofore supported the voluntary hospitals in their midst, it is surely time that the remaining 90 per cent. or a majority of them should put their hands to the plough. Let those hands make their way into the pocket and so produce a yield of funds to the hospitals which should easily and readily prove adequate to the financial necessities which largely owe their existence to the splendid work done by the hospitals during the war. This noble work has created grave liabilities which no city of honourable men can allow longer to remain existent, especially having regard to the fact, that, in the majority of the towns and cities concerned, large profits have been made by sections of the community who, we believe, it will be found, represent a very considerable percentage of the 90 per cent. of non-givers to hospitals, in the past.

In any case the time has arrived, if we are to have our hospitals financed, as they can be properly financed by June 30 next, for every hospital of any importance throughout Great Britain, and the people who reside in the district which each serves, publicly to state whether or not they intend to work with a will to save the hospitals? They cannot let them perish for want of sums which, having regard to the resources of each centre of population, are in fact relatively insignificant, compared with the actual wealth possessed, collectively, by each community enjoying a great cure-house for the sick and injured.

**See also pages 343 and 344.**

